# Ubiquitin C-terminal hydrolases cleave isopeptide- and peptide-linked ubiquitin from structured proteins but do not edit ubiquitin homopolymers

**DOI:** 10.1042/BJ20141349

**Published:** 2015-03-06

**Authors:** John S. Bett, Maria Stella Ritorto, Richard Ewan, Ellis G. Jaffray, Satpal Virdee, Jason W. Chin, Axel Knebel, Thimo Kurz, Matthias Trost, Michael H. Tatham, Ronald T. Hay

**Affiliations:** *MRC Protein Phosphorylation and Ubiquitylation Unit, The Sir James Black Centre, College of Life Sciences, University of Dundee, Dow Street, Dundee DD1 5EH, Scotland, U.K.; †Centre for Gene Regulation and Expression, College of Life Sciences, University of Dundee, Dundee DD1 5EH, Scotland, U.K.; ‡MRC Laboratory of Molecular Biology, Cambridge Biomedical Campus, Cambridge CB2 0QH, England, U.K.

**Keywords:** BAP1, SUMO, Ube2W, ubiquitin, UCH-L1, UCH-L3, UCH-L5, DUB, deubiquitylating enzyme, Ni-NTA, Ni^2+^-nitrilotriacetate, OTU, ovarian tumour, SENP, SUMO protease, Ub, ubiquitin, UCH, ubiquitin C-terminal hydrolase, USP, ubiquitin-specific protease

## Abstract

Modification of proteins with ubiquitin (Ub) occurs through a variety of topologically distinct Ub linkages, including Ube2W-mediated monoubiquitylation of N-terminal alpha amines to generate peptide-linked linear mono-Ub fusions. Protein ubiquitylation can be reversed by the action of deubiquitylating enzymes (DUBs), many of which show striking preference for particular Ub linkage types. Here, we have screened for DUBs that preferentially cleave N-terminal Ub from protein substrates but do not act on Ub homopolymers. We show that members of the Ub C-terminal hydrolase (UCH) family of DUBs demonstrate this preference for N-terminal deubiquitylating activity as they are capable of cleaving N-terminal Ub from SUMO2 and Ube2W, while displaying no activity against any of the eight Ub linkage types. Surprisingly, this ability to cleave Ub from SUMO2 was 100 times more efficient for UCH-L3 when we deleted the unstructured N-terminus of SUMO2, demonstrating that UCH enzymes can cleave Ub from structured proteins. However, UCH-L3 could also cleave chemically synthesized isopeptide-linked Ub from lysine 11 (K11) of SUMO2 with similar efficiency, demonstrating that UCH DUB activity is not limited to peptide-linked Ub. These findings advance our understanding of the specificity of the UCH family of DUBs, which are strongly implicated in cancer and neurodegeneration but whose substrate preference has remained unclear. In addition, our findings suggest that the reversal of Ube2W-mediated N-terminal ubiquitylation may be one physiological role of UCH DUBs *in vivo*.

## Abstract 2

The substrate preference of ubiquitin C-terminal hydrolase (UCH) deubiquitylating enzymes (DUBs) has been unclear. Here we report that UCH family DUBs efficiently cleave both isopeptide- and peptide-linked ubiquitin from substrates, without processing simple Ub–Ub polymers of any linkage type.

## INTRODUCTION

Conjugation of proteins with ubiquitin (Ub) is a versatile post-translational modification that regulates a number of cellular pathways and signalling events [1]. Modification of substrates is achieved through the concerted actions of a series of enzymes, starting with Ub activation by an E1 activating enzyme, transfer to any of a number of E2 conjugating enzymes, and finally substrate specificity is defined via an E3 ligase which recruits the substrate and E2 to mediate Ub transfer [2].

Conjugation of Ub to a substrate lysine (K) residue can be either a mono- or poly-ubiquitylation event, of which polyubiquitylation can occur via isopeptide Ub chains linked through any of seven internal K residues (K6, K11, K27, K29, K33, K48 and K63) [1], or linear head-to-tail peptide-linked polyUb chains [3]. In addition to these eight distinct linkage types of polymeric Ub, substrates can also be modified by monoubiquitylation of the epsilon amino group of a lysine residue or via the alpha amino group of the N-terminus of the substrate to generate a linear mono-Ub fusion protein [4–6]. An E2 conjugating enzyme responsible for the latter type of modification is Ube2W, which can monoubiquitylate the N-terminus of poly-SUMO2 when coupled with the E3 ligase RNF4 [7].

The process of Ub conjugation is regulated through the catalytic activities of deubiquitylating enzymes (DUBs), which can reverse or edit all Ub modification types to generate free monomeric Ub [8]. The ~90 DUBs in the human genome can be divided into five major classes: the ubiquitin C-terminal hydrolases (UCHs), the ubiquitin-specific proteases (USPs), the ovarian tumour (OTU) family, the Josephin domain family and the JAB1/MPN/MOV34 (JAMM) family [1,8]. The majority of DUBs show low linkage specificity, including most members of the USP family which can cleave all linkage types *in vitro* in a non-discriminatory manner [1,9]. By contrast, some DUBs are highly specific for only one type of chain linkage, for example the JAMM family member AMSH is specific for K63 linkages, OTUB1 is specific for only K48 linkages, and otulin only cleaves linear linked Ub chains [9–13]. Interestingly, some DUBs are classified as pseudoDUBs, as they contain homology with the USP superfamily but are inactive due to the lack of an active site cysteine [14,15].

The four UCH enzymes represent an unusual family of DUBs, as they are known to contain an active site cross-over loop that is thought to limit substrate accessibility [16–20]. The two smallest members of this family, UCH-L1 and UCH-L3, contain only the core UCH domain, whereas UCH-L5 and BAP1 contain the UCH domain and additional C-terminal extensions [21]. Although this class of DUB was the first to be described [22], the substrate preference of the UCH family has remained elusive. *In vitro*, purified UCH enzymes show no activity against any K-linked or linear Ub-dimers [9,23], but are able to efficiently cleave Ub from glutathione and amines such as free lysine that may form adventitiously [22,24]. In addition, UCH-L3 displays Ub hydrolase activity towards the CEP52/UBA52 Ub-ribosome precursor and Ub linearly fused to small peptides [25,26], thus it has been assumed that UCH-L1 and UCH-L3 only cleave Ub from substrates with small unstructured leaving groups [17,21]. The other two members of the UCH family UCH-L5 and BAP1 contain longer inhibitory C-terminal extensions, and are reported to be largely inactive enzymes *in vitro* until association with the proteasome and ASXL1 respectively, which relieve their autoinhibition and permit the hydrolysis of Ub isopeptides [27,28].

In the present study, we set out to identify DUBs that could preferentially cleave alpha N-terminal-linked mono-Ub from SUMO2 or Ube2W. We report that members of the UCH family are capable of efficiently cleaving peptide-linked N-terminal mono-Ub, while displaying no activity against Ub dimers of any linkage type. However, UCH DUBs could also cleave isopeptide-linked Ub from lysine 11 of SUMO2, suggesting their activity is not strictly limited to peptide-linked N-terminal Ub. Thus, the reversal of N-terminal ubiquitylation may be one physiological role of UCH DUBs *in vivo*.

## EXPERIMENTAL

### Recombinant protein purification

The DUBs USP2, UCH-L1, UCH-L3, UCH-L5 and BAP1 were purified from *Escherichia coli* as previously described [9]. The substrates 6xHis-Ub-SUMO2x4ΔN11, Ub-Ube2W, Ub-SUMO2-6xHis, Ub-SUMO2Δ1-15-6xHis, Ub-[SUMO2: 1–15]-Ub-6xHis were purified in *E. coli* Rosetta (DE3) cells with a knockout in the *ElaD* gene (gift from Rob Layfield) which encodes a bacterial DUB [29]. Bacterial cells were harvested by centrifugation and the cell pellet was resuspended in lysis buffer (50 mM Tris/HCl, 500 mM NaCl, 10 mM imidazole and, 2 mM benzamidine) (or for Ub-Ube2W lysis: 50 mM Tris/HCl, 50 mM NaCl and 2 mM benzamidine) and lysed by sonication (Digital Sonifier, Branson). Subsequently, Triton X100 was added to a final concentration of 0.5% (v/v) and the sample was centrifuged to remove any insoluble material. For Ub-SUMO2-6xHis, Ub-SUMO2Δ1-15-6xHis and Ub-[SUMO2: 1–15]-Ub-6xHis, the supernatant was filtered through a 0.2 μm filter and loaded on to a n Ni^2+^-nitrilotriacetate (Ni-NTA)–agarose (Qiagen) column and washed, eluted and dialysed overnight at 4°C against 50 mM Tris/HCl, 150 mM NaCl and 0.5 mM tris(2-carboxyethyl)phosphine (TCEP), pH 7.5. Purification of 6xHis-Ub-SUMO2x4ΔN11 was as above, followed by tobacco etch virus (TEV) protease cleavage to remove the 6xHis tag (1 mg of His-TEV protease per 100 mg of the fusion protein). Ub-SUMO2x4ΔN11 was purified by passing over an Ni-NTA–agarose column to remove His-TEV protease and 6xHis tag. Untagged Ub-Ube2W was purified using a Q Sepharose (GE Healthcare) ion-exchange column and eluted with an NaCl gradient (50-600 mM) and dialysed as above. Fractions were concentrated using Vivaspin centrifugal concentrator (Sartorius). Gel filtration chromatography on a HiLoad 16/60 Superdex 200 pg (for 6xHis-Ub-SUMO2x4ΔN11) or 75 pg column (for all other substrates) (GE Healthcare) was carried out as a final purification step. Ub-K11-SUMO2 and Ub dimers (K6, K27, K29 and K33) were synthesized as previously described [9,30,31]. Linear Ub dimers linked via the peptide bond were expressed as GST-fusion proteins in bacteria. The GST-tag was removed with Prescission Protease and the dimers were purified over a Source 15 S column and concentrated using Vivaspin 5 kDa molecular-mass cut-off filters (Sartorius). K48-linked Ub dimers were made enzymatically using UBE1 and GST–UBE2K. The K63 Ub dimers were also made enzymatically using UBE1 and UBE2N and UBE2V1. The enzymes were removed by ion-exchange chromatography and the dimers were purified using a Source 15 S column.

### Ubiquitylation, deubiquitylation, immunodetection and quantification

*In vitro* ubiquitylation of Ub-SUMO2x4ΔN11 was carried out at 37°C for 1 h as described previously [7]. Ubiquitylated Ub-SUMO2x4ΔN11 was resolved from the ubiquitylation reaction and chains purified by gel filtration on Superdex 75 medium (GE Healthcare). For DUB assays visualized by Coomassie Blue-stained gels, 2 μg of substrate was incubated with 100 ng/μl DUB (1.38 μM GST-USP2 3.7 μM 6xHis-UCHL1, 1.85 μM GST-UCHL3, 1.5 μM GST-UCHL5 or 0.9 μM GST-BAP1 at 37°C for 1 h in a total volume of 10 μl in assay buffer (50 mM Tris/HCl pH 7.6 and 5 mM DTT). Reaction products resolved on 4–12% NuPage Bis-Tris gradient gels in MES buffer (Life Technologies) and subsequently visualized by Coomassie Blue-staining. Immunoblotting was carried out using anti-Ub (DAKO) and anti-SUMO2 antibodies [32]. Densitometry analysis of gel images was carried out using ImageJ (version 1.47; NIH). For each protease assay, the extent of substrate cleavage was determined and represented as a percentage of the control reaction (USP2). A heat map presenting these data was generated using Perseus (available from http://www.perseus-framework.org/). Ub-rhodamine-110-Gly assays were carried out as previously described [9].

### MALDI-TOF DUB assay for establishing kinetic parameters

For the analysis by MALDI-TOF DUB assay, bovine serum albumin (BSA), Tris and DTT were purchased from Sigma–Aldrich. MALDI-TOF MS materials (targets, matrix and protein calibration mixture) were from Bruker Daltonics. Screening for activity and specificity of UCHL1, UCHL3, UCHL5 and BAP1 against Ub-SUMO2, Ub-Ube2W, Ub-4xSUMO2ΔN11, Ub-SUMO2Δ1-15 and Ub-K11-SUMO2 was performed as previously described [9]. Briefly, each UCHL family member was incubated at different concentrations (0.02, 0.2, 2, 20 and 200 ng/μl) with each substrate (30 ng/μl). Both enzymes and substrates were freshly prepared in the reaction buffer (40 mM Tris/HCl, pH 7.6, 5 mM DTT, and 0.005% BSA) for each run. The enzymes were pre-incubated in the reaction buffer for 10 min at 30°C; afterwards, the substrates were added and the reaction mixture was incubated for 60 min at 30°C. The reaction was stopped by adding trifluoroacetic acid (TFA) to a final concentration of 2% (v/v). Possible background due to contamination of the substrate with Ub monomers was measured in a reaction buffer in which the enzyme was excluded and Ub intensities were normalized accordingly. The kinetic constants of each enzyme were determined using the MALDI-TOF DUB assay. For calculation of *k*_m_ and *V*_max_, each enzyme concentration was chosen so that the reaction was linear with a molar excess of the specific substrate over 60 min at 30°C (shaking at 850 rpm). Substrates were chosen according to detected activity (>10%) against the DUB. All data were plotted by SigmaPlot (version 12.5), using the enzyme kinetics tool and the following parameters: Single Substrate Study, Michaelis–Menten equation=*V*_max_ × *S*/(*k*_m_ + *S*). The steady state of reactions were determined by incubating a fixed amount of enzymes (7.30 μM UCHL1, 3.82 μM UCHL3, 0.03 μM UCHL5 and 1.87 μM BAP1) with the indicated excess of substrate.

## RESULTS

### UCH DUBs possess peptide-linked N-terminal deubiquitylating activity

**Figure 1:**
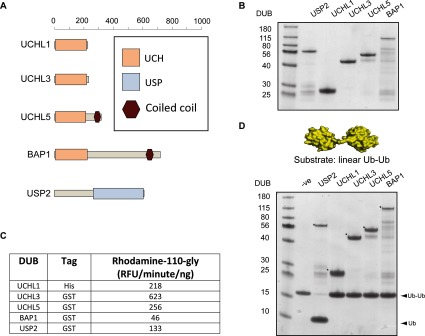
The UCH family of DUBs cleave Ub-rhodamine-110-Gly but are inactive towards Ub dimers (**A**) Domain maps of each of the UCH DUBs and the broadly active positive control USP2. (**B**) A 1 μg amount of each DUB was fractionated by SDS/PAGE and visualized by Coomassie Blue staining. (**C**) DUB activity assessed against the artificial substrate Ub-rhodamine-110-Gly. (**D**) Structure of linear Ub–Ub, PDBL 2W9N [23]. DUBs were incubated with linear Ub–Ub dimers and the reaction products were fractionated by SDS/PAGE and visualized by Coomassie Blue staining. Asterisks (*) denote the DUB in each reaction. DUB assays were carried out for 1 h at 37°C.

**Figure 2:**
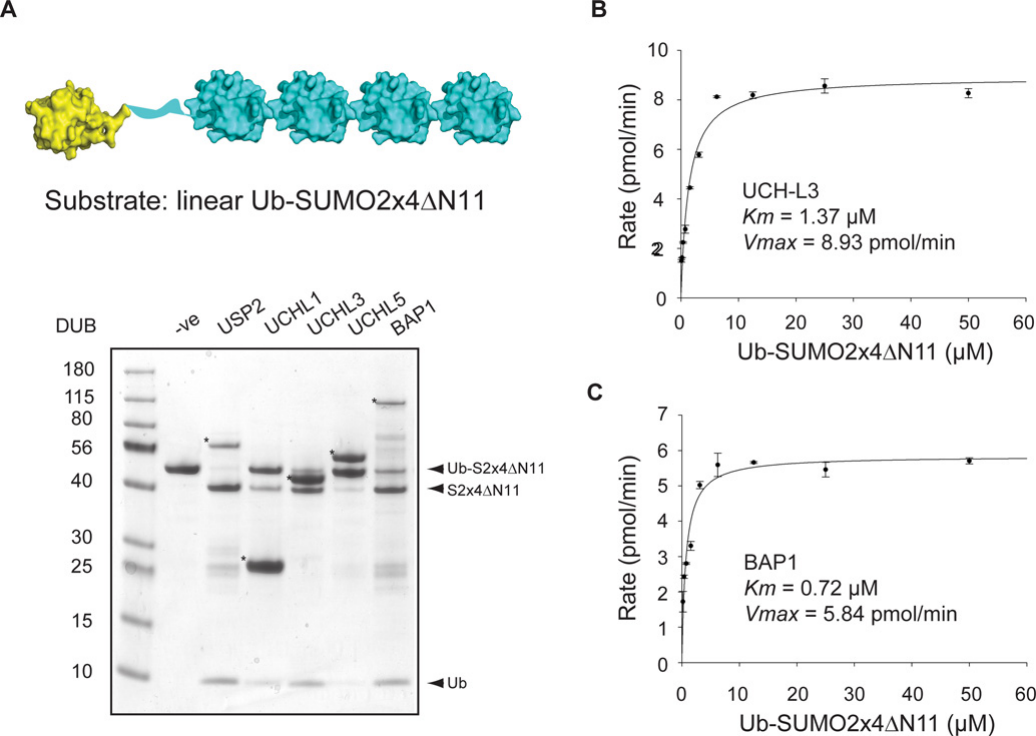
The UCH DUBs can cleave Ub from the N-terminus of polySUMO2 (**A**) Structural representation of the Ub-SUMO2x4ΔN11 substrate was created by using the Ub structure (PDB: 1UBQ, yellow) [46] and the SUMO2 structure (PDB: 1WM2, blue) [47] (the flexible N-terminus of SUMO2 is represented by a blue ribbon). DUBs were incubated with Ub-SUMO2x4ΔN11 and the reaction products were fractionated by SDS/PAGE and visualized by Coomassie Blue staining. Asterisks (*) denote the DUB in each reaction (**B**) Michaelis–Menten kinetic analysis of UCH-L3 with Ub-SUMO2x4ΔN11 as substrate. (**C**) Michaelis–Menten kinetic analysis of BAP1 with Ub-SUMO2x4ΔN11 as substrate.

The UCH family of DUBs (UCH-L1, UCH-L3, UCH-L5 and BAP1) (Figure 1A) are reported to be inactive against Ub dimers of all eight linkage types [9], and therefore we reasoned that they would be suitable candidates as DUBs that preferentially cleave N-terminally-linked Ub. To test this, full-length versions of all four UCH DUBs were first expressed and purified from bacteria and visualized by Coomassie Blue-stained gel (Figure 1B). All four UCH enzymes were active against the generic substrate Ub-rhodamine-110-Gly (Figure 1C), but were not capable of cleaving linear Ub dimers (Ub–Ub), while the positive control USP2 completely cleaved this substrate (Figure 1D). To confirm that the UCH enzymes do not act upon Ub isopeptides of any type, Ub dimers of all linkages (K6, K11, K27, K29, K33, K48 and K63) were used as substrates against the four members of the UCH family. USP2 was able to fully hydrolyse all Ub dimers into free Ub, with the exception of K27 against which it showed reduced activity (Supplementary Figure S1). However, the UCH family enzymes were unable to hydrolyse any of the Ub linkage types, apart from a low level activity of UCH-L3, UCH-L5 and BAP1 against K11-linked dimers (Supplementary Figure S1). To determine whether any of the UCH DUBs could cleave Ub from SUMO2, we used a linear fusion of Ub to four tandem copies of SUMO2 (Ub-SUMO2x4ΔN11), the result of Ube2W/RNF4-mediated N-terminal ubiquitylation of polySUMO2 (Figure 2A) [7]. Both UCH-L3 and BAP1 utilized this substrate generating free Ub and SUMO2x4ΔN11, while UCH-L1 and UCH-L5 displayed lower activity (Figure 2A). To quantitatively evaluate the ability of UCH DUBs to cleave N-terminal Ub from polySUMO2, we used a recently established assay that monitors the release of free Ub by a MALDI-TOF DUB assay [9]. Michaelis–Menten kinetics were established for UCH-L3 (Figure 2B) and BAP1 (Figure 2C) against Ub-SUMO2x4ΔN11, and the catalytic efficiencies (*k*_cat_/*K*_m_) were calculated to be 5.76×10^3^ M^−1^·s^−1^ for UCH-L3 and 1.45×10^4^ M^−1^·s^−1^ for BAP1 (Table 1). Therefore, UCH family members possess efficient N-terminal deubiquitylation activity while remaining inactive towards Ub dimers of all linkage types.

**Table 1:** Steady-state kinetic parameters for UCH family members

	Enzyme	*K*_m_ (μM)	*V*_max_ (pmol·min^−1^)	*k*_cat_ (s^−1^)	*k*_cat_/*K*_m_ (M^−1^·s^−1^)
Ub-SUMO2					
	UCHL1	5.22±0.49	0.03152	0.0000136	2.60
	UCHL3	4.94±0.70	0.2	0.000192	3.89×10^1^
Ub-4xS2ΔN11					
	UCHL3	1.37±0.14	8.93	0.00789	5.76×10^3^
	BAP1	0.72±0.09	5.84	0.0104	1.45×10^4^
Ub-Ube2W					
	UCHL3	1.58±0.14	7.13	0.00630	3.99×10^3^
	UCHL5	6.30±0.93	0.9	0.10	1.59×10^4^
	BAP1	1.34±0.20	6.65	0.0119	8.86×10^3^
Ub-(Δ 1–15) SUMO2					
	UCHL3	0.29±0.05	1.27	0.00112	3.89×10^3^
Ub-K11-SUMO2					
	UCHL3	0.51±0.09	1.23	0.00107	2.08×10^3^

### UCH family DUBs cleave Ub chains *en bloc* from polyubiquitylated linear Ub-SUMO2x4ΔN11

**Figure 3:**
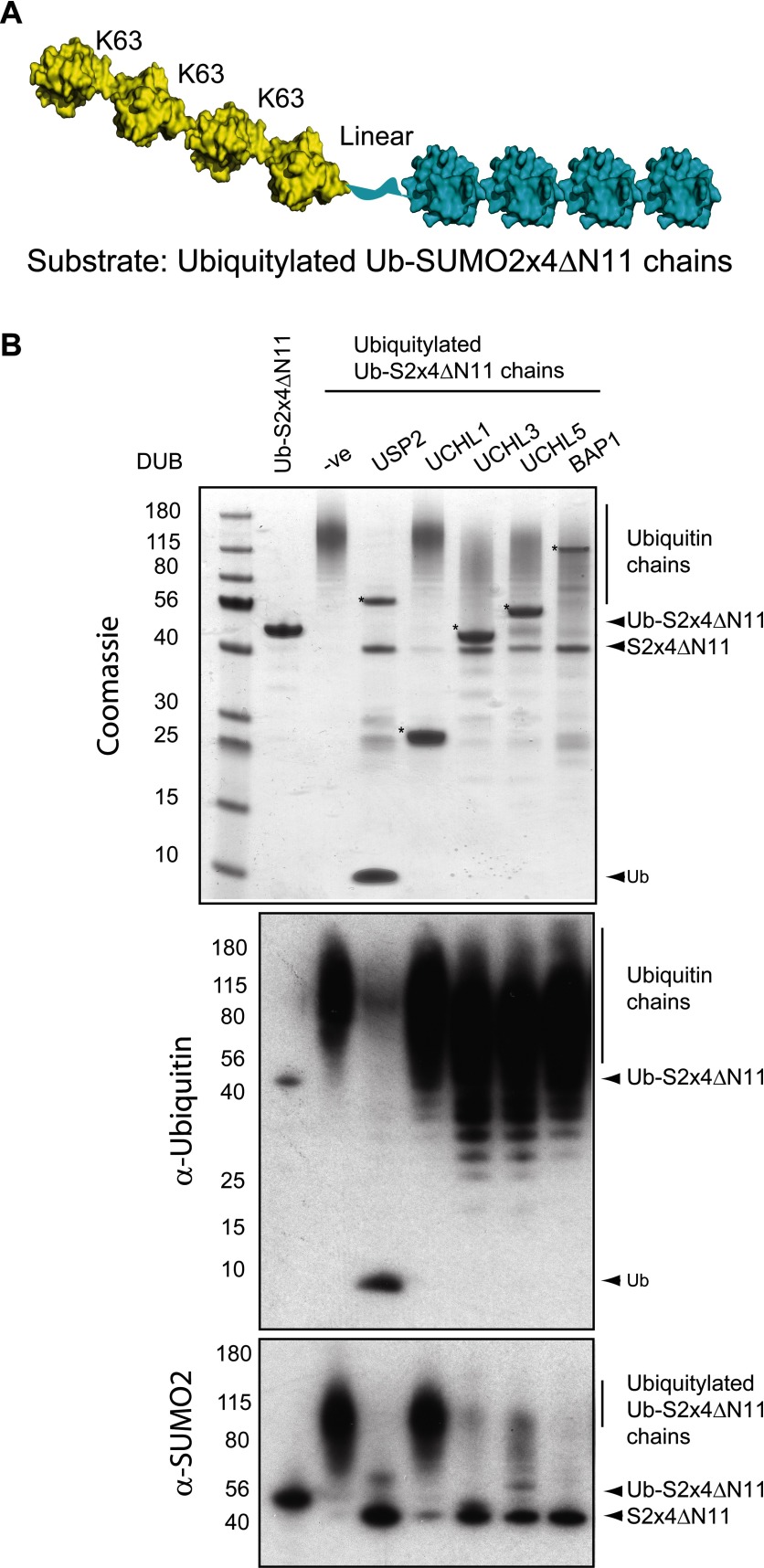
UCH DUBs cleave ubiquitin chains from polyUb-SUMO2x4ΔN11 *en bloc* (**A**) Structural representation of the K63-polyubiquitylated substrate. K63-linked Ub chains (PDB: 2JF5, yellow) [23] and SUMO2 (PDB: 1WM2, blue) [47] (the flexible N-terminus of SUMO2 is represented by a blue ribbon). (**B**) K63 polyub linked to Ub-SUMO2x4ΔN11 was incubated with the indicated DUBs and the reaction products were fractionated by SDS/PAGE. Reaction products were visualized by Coomassie Blue staining (upper panel), Western blotting with an antibody recognizing ubiquitin (middle panel) and Western blotting with an antibody to SUMO-2 (lower panel). Asterisks (*) denote the DUB in each reaction.

As substrates containing longer ubiquitylated chains more closely represent physiological substrates than Ub dimers, we determined how such substrates were utilized by UCH DUBs. To generate substrate containing long Ub chains, Ub-SUMO2x4ΔN11 was ubiquitylated *in vitro* using RNF4 and Ube2N/Ube2V1, which generates K63-linked Ub chains on the N-terminally linked Ub moiety [7] (Figure 3A). Ubiquitylated Ub-SUMO2x4ΔN11 chains were purified by gel filtration (Supplementary Figure S2) and used as substrate against the UCH DUBs. Reaction products were visualized either by Coomassie Blue staining (Figure 3B, upper panel) or by immunoblotting with antibodies to either Ub (Figure 3B, middle panel) or SUMO2 (Figure 3B, lower panel). Positive control USP2 cleaved all Ub linkages, resulting in the appearance of unmodified SUMO2x4ΔN11 substrate (Figure 3B, top and bottom panels) and monomeric Ub (Figure 3B, top and middle panels). However, UCH-L3, UCH-L5, BAP1 (and to a lesser extent UCH-L1) treatment resulted in the release of SUMO2x4ΔN11 (Figure 3B, top and bottom panels) in the absence of monomeric Ub release (Figure 3B, top and middle panels). Thus, UCH DUBs can cleave the N-terminal Ub and attached chain from Ub-SUMO2x4ΔN11 *en bloc*, but do not exert any Ub hydrolase activity towards the chains themselves.

### Characterizing UCH-mediated cleavage of peptide-linked Ub-Ube2W

**Figure 4:**
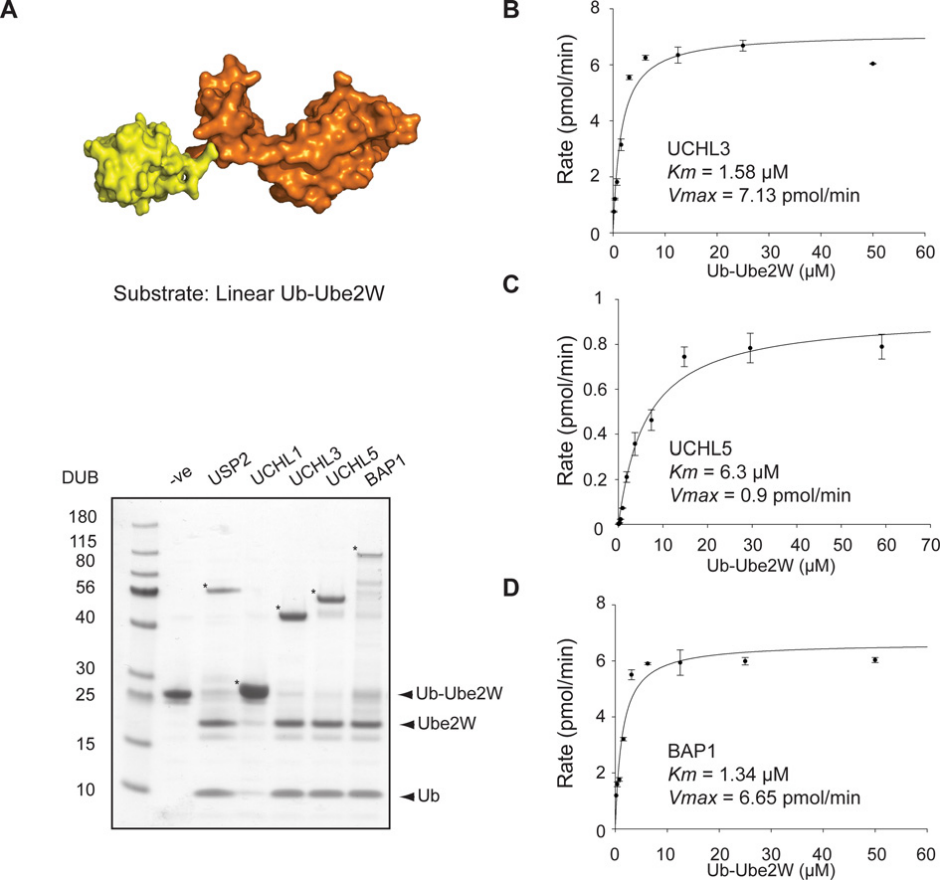
UCH mediated N-terminal deubiquitylation of Ub-Ube2W (**A**) Structural representation of Ub-Ube2W substrate. Ub (PDB: 1UBQ, yellow) [46] and Ube2W (PDB: 2A7L, gold) [48]. Ub-Ube2W was incubated with the indicated UCH DUB or positive control USP2 and the reaction products were fractionated by SDS/PAGE and visualized by Coomassie Blue staining. Asterisks (*) denote the DUB in each reaction. Michaelis–Menten kinetic analysis of UCH-L3 (**B**), UCH-L5 (**C**) and BAP1 (**D**) using Ub-Ube2W as substrate.

To determine whether the ability to cleave N-terminal Ub was a general property of the UCH family, we characterized DUB activity using a second substrate. We expressed and purified a linear fusion of Ub to Ube2W (Ub-Ube2W), the product of Ube2W N-terminal auto-ubiquitylation [7]. UCH-L3, UCH-L5, BAP1 and positive control USP2 completely cleaved Ub from Ube2W, as visualized by the release of free Ub and Ube2W on a Coomassie Blue-stained gel (Figure 4A), while UCH-L1 had a lower activity against this substrate (Figure 4A). To quantitatively establish enzymatic parameters, we used the MALDI-TOF DUB assay to determine Michaelis–Menten kinetics against Ub-Ube2W for UCH-L3 (Figure 4B), UCH-L5 (Figure 4C) and BAP1 (Figure 4D). The catalytic efficiency (*k*_cat_/*K*_m_) against Ub-Ube2W was 3.99×10^3^ M^−1^·s^−1^ for UCH-L3, 8.86×10^3^ M^−1^·s^−1^ for BAP1, and the highest efficiency was 1.59×10^4^ M^−1^·s^−1^ for UCH-L5 (Table 1). Therefore, UCH-L3, UCH-L5 and BAP1 possess a general N-terminal deubiquitylation activity.

### Requirements of UCH-mediated Ub cleavage from monomeric SUMO2

**Figure 5:**
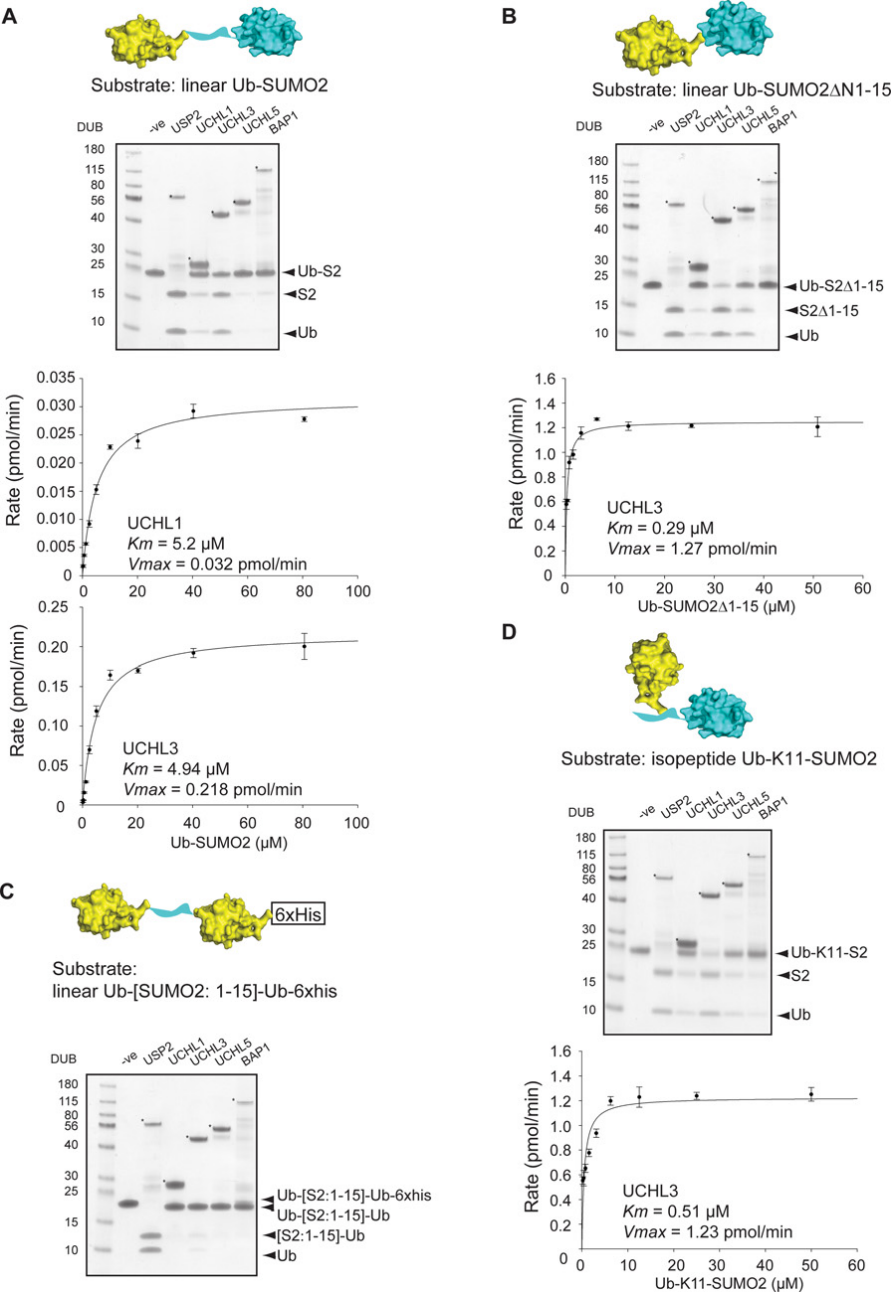
Ub-SUMO2 substrate requirements for cleavage with UCH enzymes (**A**) Linear Ub-SUMO2 substrate (structural representation above) was incubated with UCH DUBs and the reaction products were fractionated by SDS/PAGE and visualized by Coomassie Blue staining (top panel). Michaelis–Menten kinetic analysis of UCH-L1 and UCH-L3 with Ub-SUMO2 as substrate (bottom panels). (**B**) Structural representation of Ub-SUMO2ΔN1-15 used as substrate. DUB activity was assessed as in (**A**). Michaelis–Menten kinetic analysis of UCH-L3 with Ub-SUMO2ΔN1-15 as substrate. (**C**) Structural representation of Ub-[SUMO2 1:15]-Ub-6xHis used as substrate. DUB activity was assessed by release of free Ub and free [SUMO2:1-15]-Ub or cleavage of the 6-His tag. (**D**) Structural representation of isopeptide-linked Ub-K11-SUMO2 used as substrate. DUB activity was assessed as in (**A**). Michaelis–Menten kinetic analysis of UCH-L3 with Ub-K11-SUMO2 as substrate. Asterisks (*) denote the DUB in each reaction. Structural representation of each Ub-SUMO2 substrate was created by using the Ub structure (PDB: 1UBQ, yellow) [46] and the SUMO2 structure (PDB: 1WM2, blue) [47] (the flexible N-terminus of SUMO2 is represented by a blue ribbon).

To further investigate UCH-mediated cleavage of Ub from SUMO2, we generated a series of Ub-SUMO2 dimers to determine UCH enzyme requirements for this reaction. Linear Ub-SUMO2 dimers were purified and used as substrate in DUB assays (Figure 5A), and cleavage was assessed by the release of free Ub and free SUMO2 on Coomassie Blue-stained gels. UCH-L1 and UCH-L3 displayed the greatest activity against Ub-SUMO2 dimers, while UCH-L5 and BAP1 activity was limited. We established Michaelis–Menten kinetics of UCH-L1 and UCH-L3 against Ub-SUMO2 dimers by the MALDI-TOF DUB assay (Figure 5A), and the catalytic efficiency (*k*_cat_/*K*_m_) of UCHL1 and UCHL3 against Ub-SUMO2 dimers were calculated as 2.6 M^−1^·s^−1^ and 3.89×10^1^ M^−1^·s^−1^ respectively (Table 1). Since UCH DUBs cleave Ub from SUMO2 which contains an unstructured N-terminus, but do not cleave structurally compact Ub dimers, we wanted to determine the contribution of the structural flexibility of the Ub conjugation site to cleavage by UCH DUBs. Therefore, we deleted the flexible N-terminal 15 residues of SUMO2 and expressed a linear Ub-SUMO2ΔN1-15 dimer, which comprises the Ub globular domain fused directly to the SUMO2 globular domain (Figure 5B). Surprisingly, with the exception of BAP1, we found that the UCH family all displayed activity against this substrate (Figure 5B). Michaelis–Menten kinetics were established for UCH-L3 against Ub-SUMO2ΔN1-15 by MALDI-TOF (Figure 5B). Strikingly, the catalytic efficiency (*k*_cat_/*K*_m_) for UCHL3 against Ub-SUMO2ΔN1-15 was 3.89×10^3^ M^−1^·s^−1^, which is approximately 100-fold higher than the efficiency of UCH-L3 against wild type Ub-SUMO2 dimers (Table 1). This demonstrates that UCH enzymes can cleave Ub from structured proteins, and suggests that structural inflexibility does not explain why UCH enzymes do not cleave Ub dimers. To explore this further, we created Ub dimers separated by a flexible linker by inserting residues 1–15 from SUMO2 between two Ub monomers to create a linear Ub-[SUMO2: 1–15]-Ub dimer with a C-terminal 6xHis tag. Interestingly, the UCH DUBs showed little or no ability to cleave after the first Ub, but were capable of completely cleaving the 6xHis tag off the second Ub (Figure 5C) (Supplementary Figure S3). Thus, UCH family DUBs can cleave Ub from structured conjugation sites, and the close proximity of the protomers in Ub dimers does not explain the inability of UCH enzymes to cleave them. Finally, to determine whether UCH enzymes possessed Ub isopeptidase activity in addition to N-terminal peptide deubiquitylating activity, we used a chemically synthesized isopeptide-linked Ub-SUMO2, where SUMO2 had been site-specifically modified with Ub on lysine 11 [31] (Ub-K11-SUMO2). Interestingly, all of the UCH family members and particularly UCH-L3 showed activity against this substrate (Figure 5D). Michaelis–Menten enzyme kinetics were established for UCH-L3 against Ub-K11-SUMO2 (Figure 5D) and the catalytic efficiency (*k*_cat_/*K*_m_) was 2.11×10^3^ M^−1^·s^−1^, similar to that of UCH-L3 against Ub-SUMO2ΔN1-15 (Table 1). Thus, UCH family DUBs can efficiently cleave both isopeptide and peptide Ub-substrate bonds, but not simple Ub–Ub bonds.

## DISCUSSION

**Figure 6:**
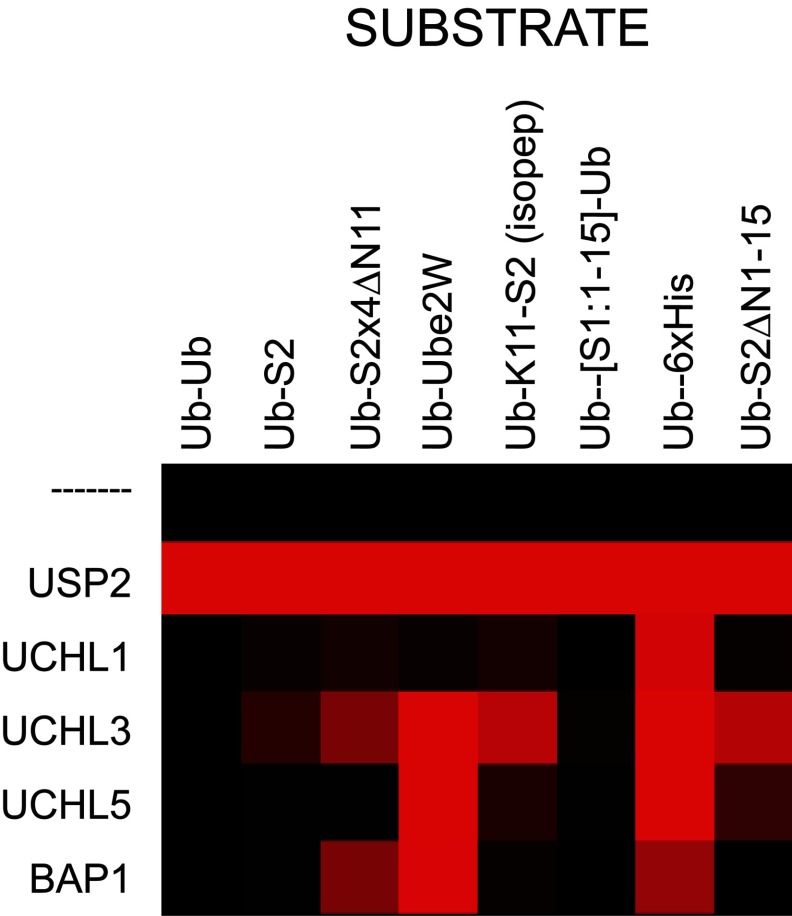
Heat map of UCH DUB activity All DUB activity is normalized to positive control USP2 and the activity of each UCH DUB is displayed relative to this. The scale is from red (100% activity) to black (0% activity).

In biochemical assays, the UCH family of DUBs display no protease activity towards Ub dimers or tetramers no matter the linkage type (Supplementary Figure S1) [9,23]. It has been known for some time that UCH-L1 and UCH-L3 can cleave small adducts of Ub such as glutathione and free lysine [22,24], and display low activity against ubiquitylated protein substrates [33]. This has led to the suggestion that their function *in vivo* is to regenerate free Ub from adventitiously generated adducts [8,21]. In addition, UCH-L5 and BAP1 are known to be inactive towards Ub–Ub linkages in the absence of their cellular cofactors [27,28]. We have demonstrated that the UCH DUBs display ability to cleave N-terminally linked and isopeptide-bond-linked Ub from a number of protein constructs *in vitro*. The results are summarized in the heat map in Figure 6. These findings imply that UCH enzymes are specifically averse to cleaving Ub from Ub in a simple chain, but that, with the exception of UCH-L1, they are certainly capable of removal of Ub from large protein substrates. N-terminal ubiquitylation has been described *in vivo* [4–6], and the E2 conjugating enzyme Ube2W is capable of synthesizing peptide bond-linked substrates *in vitro* [7]. This opens the possibility that one function of UCH proteases is to cleave peptide-bond-linked Ub. However, the fact that UCH enzymes possess low activity towards Ub–Ub peptide linkages indicates that the modified protein cannot be Ub itself.

Kinetic analysis revealed that UCH-L3 (and to a lesser extent UCH-L1) is capable of cleaving N-terminal peptide-linked Ub from SUMO2x4ΔN11 and Ube2W with a *k*_cat_/*K*_m_ of 5.76×10^3^ M^−1^·s^−1^ and 3.99×10^3^ M^−1^·s^−1^ respectively. This is several orders of magnitude lower than what has been reported for the yeast UCH enzyme YUH1 against the artificial substrate Ub-AMC (2.23×10^8^ M^−1^·s^−1^) [17], but is in the range of what has been reported for the OTU family DUB TRABID to cleave K63 and K29 chains: 2.5×10^3^ M^−1^·s^−1^and 1×10^5^ M^−1^·s^−1^ respectively [30]. The observation that UCH enzymes can cleave N-terminal mono-Ub from SUMO2 and Ube2W is in keeping with previous reports showing that UCH-L3 has some activity towards the Ub precursor protein Ub-CEP52, and a variety of fusions of Ub to short peptides including the mutant Ub UBB+1 [25,26,34]. It is striking that when we ubiquitylated Ub-SUMO2x4ΔN11 using RNF4 and Ube2N/Ube2V1 which attaches K63-linked chains on to the proximal Ub [7], UCH-L3, UCH-L5 and BAP1 exclusively cleave the N-terminal linked Ub to remove the K63 chains *en bloc*, completely unprocessed.

Structural studies of UCH family enzymes either in isolation or in complex with Ub suicide probes have revealed a dynamic active site cross-over loop that has been predicted to prevent cleavage of Ub if conjugated to structured regions of protein [16–20,35]. This is because it has been often assumed that the leaving group must pass through the narrow loop, which for UCH-L3 is limited to around 15 Å (1 Å=0.1 nm) and is thus too small to allow a structured protein to pass [17]. Extension of this loop can render UCH-L3 capable of hydrolysing Ub K48- and K63-linked dimers, supporting a restrictive role of the loop in allowing structured substrates access to the active site [36]. However, it seems improbable that the leaving group could be fed completely through this loop, given that we observe efficient removal of Ub from the N-terminus of large, structured proteins. It is also interesting to note that UCH enzymes would not hydrolyse Ub–Ub dimers where we inserted the flexible 1–15 N-terminal residues of SUMO2, suggesting that Ub dimers may be intrinsically inhibitory to UCH family DUBs. In agreement with this idea, it has been previously reported that K48 and K63 Ub dimers can inhibit the cleavage of Ub-AMC by both UCHL1 and UCHL3 [37]. One possibility is that because the active site cross-over loop is actually conformationally flexible, in some crystal structures the loop may be stabilized in the ‘closed’ conformation. This flexibility could allow access to substrates such as Ub-SUMO2 or Ub-Ube2W to the active site when the loop is in the unstructured ‘open’ conformation. However, binding of diubiquitin may induce a conformational change that stabilizes the loop in the ‘closed’ conformation. A similar situation is evident in the case of the SUMO proteases (SENPs). Crystal structures of SENP1 and SENP2 both with and without substrates [38–41] reveal that the active sites of these enzymes are occluded with an aromatic side chain acting as a ‘lid’ to close the catalytic channel. However, recent NMR analysis on SENP1 has revealed that Trp-465, the aromatic residue constituting the ‘lid’ is highly mobile in solution, thus allowing substrates to access the active site [42]. Structural studies of UCH-L3 in complex with either linear Ub–Ub or Ub-SUMO2x4ΔN11 will be beneficial in helping to resolve these questions.

Our observation that UCH-L5 and BAP1 both efficiently cleave N-terminal Ub from Ub-Ube2W and Ub-SUMO2x4ΔN11 was unexpected. While individual UCH domains from UCH-L5 and BAP1 display activity towards K48 Ub dimers [43], full-length enzymes do not cleave isopeptide-linked Ub unless associated with their cofactors, the proteasome and ASXL1 respectively [27,28,44]. Indeed, we find that both UCH-L5 and BAP1 in isolation are inactive against Ub–Ub dimers of all linkage types, but can efficiently cleave Ub-Ube2W with *k*_cat_/*K*_m_ values of 1.59×10^4^ M^−1^·s^−1^ and 8.86×10^3^ M^−1^·s^−1^ respectively. Interestingly, BAP1 cleaves Ub fused to four copies of SUMO2 more efficiently than it cleaves Ub-SUMO2 dimers, and while UCH-L5 showed little activity towards Ub fusions to SUMO2, it efficiently cleaves Ub-Ube2W. This suggests that multiple factors in addition to the presence of N-terminal mono-Ub influence DUB activity. Interestingly, as UCH-L5 is a proteasome-associated DUB, one function may be to remove N-terminal Ub from substrates that are targeted to the proteasome by the Ub-fusion degradation (UFD) pathway [45].

In summary, we have demonstrated that the UCH family members efficiently cleave isopeptide- and peptide-linked Ub from substrates but are inactive towards unbranched Ub polymers. Despite intense research into UCH family enzymes due to their links to neurodegenerative disease and cancer, the substrate preference of this DUB class have remained largely elusive. A greater understanding of the role and nature of N-terminally ubiquitinated proteins may have bearing upon our understanding of these functionally elusive proteases.

## Online data

Supplementary data
