# Supplementary material for: Ubiquitin C-terminal hydrolases cleave isopeptide- and peptide-linked ubiquitin from structured proteins but do not edit ubiquitin homopolymers
Source: Biochem J. 2015 Mar 6;466(Pt 3):489–98. doi: 10.1042/BJ20141349 (PMC4353193; doi:10.1042/BJ20141349)
Supplement: Supplementary data [file bj4660489ntsadd.pdf]

SUPPLEMENTARY FIGURES

FIGURE S1

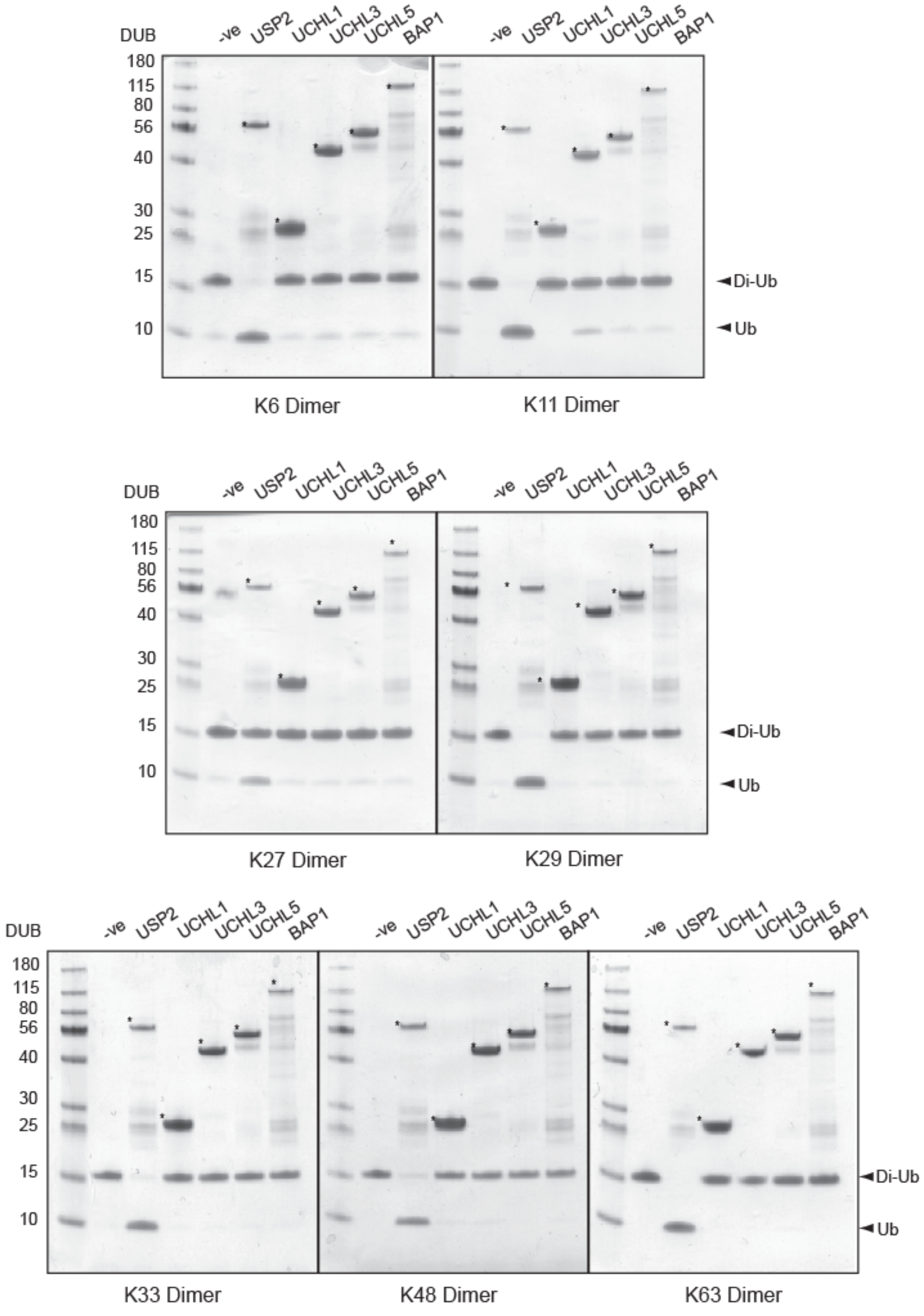

FIGURE S1. Each of the 7 indicated Ub-Ub isopeptide-linked dimers were used as substrate with the UCH DUBs or positive control USP2 and reaction products fractionated by SDS-PAGE and visualised on Coomassie-stained gels. UCH DUBs are unable to cleave any Ub-Ub dimer apart from weak activity towards K11 dimers. USP2 cleaves all dimers, but has lower activity towards K27 dimers. Asterisks (*) denote the DUB in each reaction on Coomassie stained gels.

Figure S2

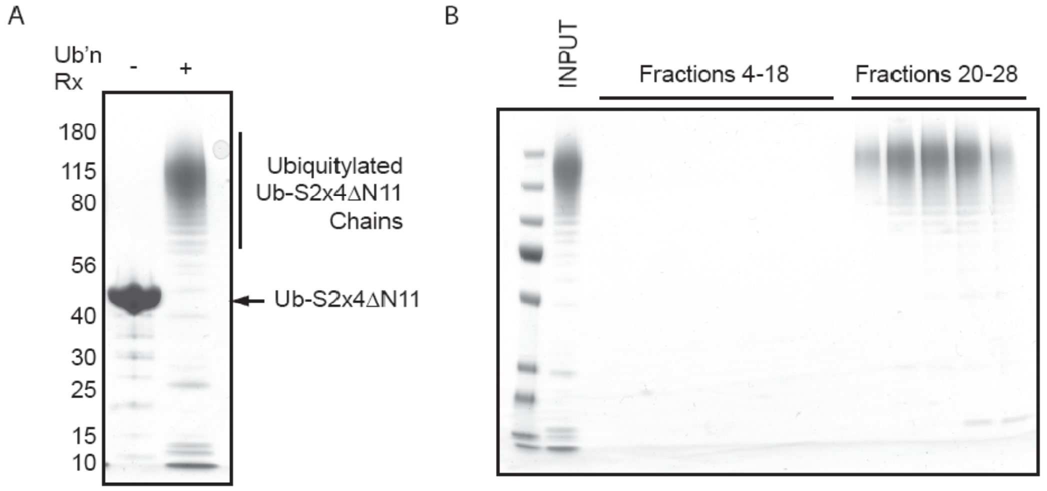

FIGURE S2. (A) Ub-SUMO2x4ΔN11 was ubiquitylated *in vitro* in a reaction containing the E2 pair Ube2N/Ube2V1 and the E3 RNF4. (B) The *in vitro* ubiquitylation reaction was resolved by gel filtration to separate polyUb-SUMO2x4ΔN11 from free Ub and other components of the ubiquitylation assay.

Figure S3

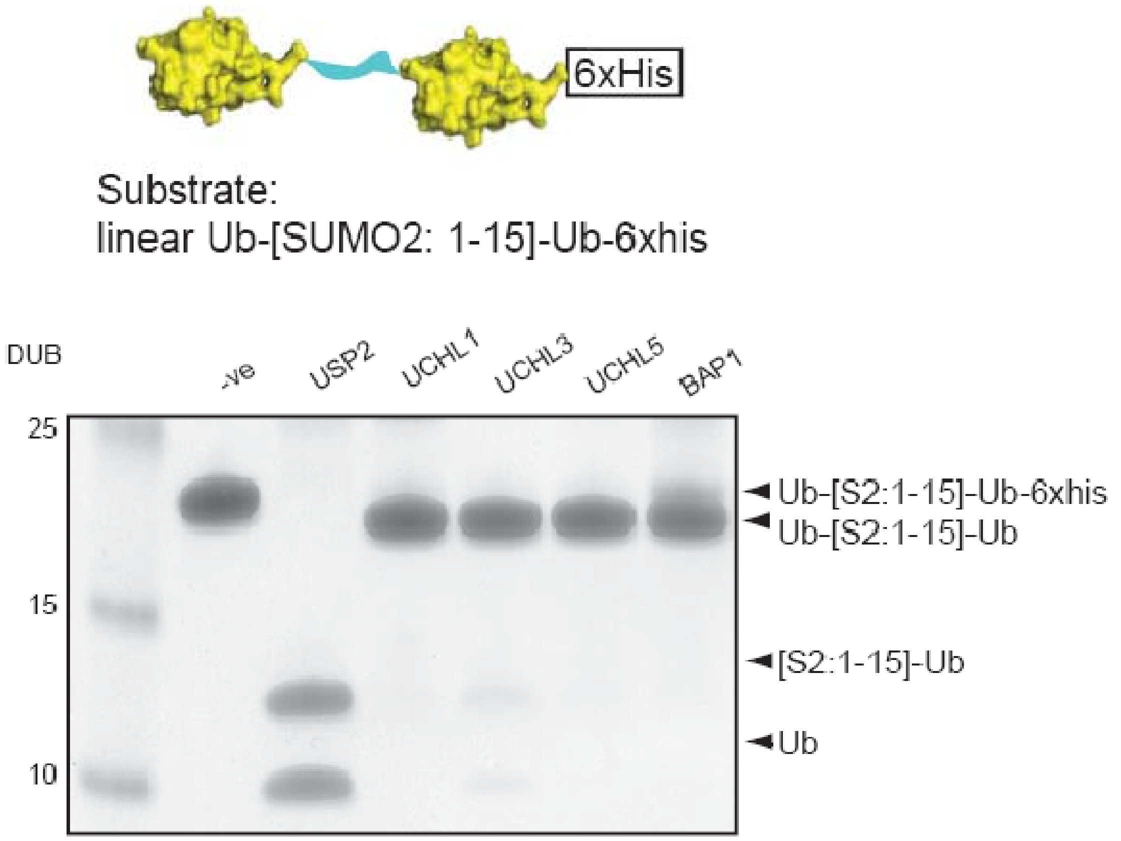

FIGURE S3. Enlarged Version of Figure 5C showing the efficient removal of the C-terminal 6xHis tag from Ub-[SUMO2: 1-15]-Ub by UCH DUBs.

Supplemental File: Protein Sequences of DUBs and Substrates

Ub-4xSUMO2∆N11

GAMGMQIFVKTLTGKTITLEVEPSDTIENVKAKIQDKEGIPPDQQRLIFAGKQLEDGRTLSDY
NIQKESTLHLVLRLRGGGSEEKPKEGVKTENDHINLKVAGQDGSVVQFKIKRHTPLSKLMKA
YCERQGLSMRQIRFRFDGQPINETDTPAQLEMEDEDTIDVFQQQTGGGSTENDHINLKVAG
QDGSVVQFKIKRHTPLSKLMKAYCERQGLSMRQIRFRFDGQPINETDTPAQLEMEDEDTID
VFQQQTGGGSTENDHINLKVAGQDGSVVQFKIKRHTPLSKLMKAYCERQGLSMRQIRFRF
DGQPINETDTPAQLEMEDEDTIDVFQQQTGGGSTENDHINLKVAGQDGSVVQFKIKRHTPL
SKLMKAYCERQGLSMRQIRFRFDGQPINETDTPAQLEMEDEDTIDVFQQQTGG

Ub-Ube2W

MQIFVKTLTGKTITLEVEPSDTIENVKAKIQDKEGIPPDQQRLIFAGKQLEDGRTLSDYNIQKE
STLHLVLRLRGGASMQTTGRRVEVWFPKRLQKELLALQNDPPPGMTLNEKSVQNSITQWIV
DMEGAPGTLYEGEKFQLLFKFSSRYPFDSPQVMFTGENIPVHPHVYSNGHICLSILTEDWS
PALSVQSVCLSIISMLSSCKEKRRPPDNSFYVRTCNKNPKKTKWWYHDDTC*

Ub-SUMO2

MQIFVKTLTGKTITLEVEPSDTIENVKAKIQDKEGIPPDQQRLIFAGKQL
EDGRTLSDYNIQKESTLHLVLRLRGGMSEEKPKEGVKTENDHINLKVAGQ
DGSVVQFKIKRHTPLSKLMKAYCERQGLSMRQIRFRFDGQPINETDTPAQ
LEMEDEDTIDVFQQQTGGHHHHHH*

Ub-SUMO2∆N1-15

MQIFVKTLTGKTITLEVEPSDTIENVKAKIQDKEGIPPDQQRLIFAGKQLEDGRTLSDYNIQKE
STLHLVLRLRGGHINLKVAGQDGSVVQFKIKRHTPLSKLMKAYCERQGLSMRQIRFRFDGQ
PINETDTPAQLEMEDEDTIDVFQQHTGGHHHHHH*

Ub-[SUMO2:1-15]-Ub

MQIFVKTLTGKTITLEVEPSDTIENVKAKIQDKEGIPPDQQRLIFAGKQLEDGRTLSDYNIQKE
STLHLVLRLRGGMSEEKPKEGVKTENDMQIFVKTLTGKTITLEVEPSDTIENVKAKIQDKEGI
PPDQQRLIFAGKQLEDGRTLSDYNIQKESTLHLVLRLRGGHHHHHH*

Ub-K11-SUMO2

(SUMO2)

MSEEKPKEGVKTENDHINLKVAGQDGSVVQFKIKRHTPLSKLMKAYAERQGLSMRQIRFRF
DGQPINETDTPAQLEMEDEDTIDVFQQQTGGHHHHHH

(Ubiquitin)

MQIFVKTLTGKTITLEVEPSDTIENVKAKIQDKEGIPPDQQRLIFAGKQLEDGRTLSDYNIQKE
STLHLVLRLRGG

GST-USP2

MSPILGYWKIKGLVQPTRLLLEYLEEKYEEHLYERDEGDKWRNKKFELGLEFPNLPYYIDGD
VKLTQSMAIIRYIADKHNMLGGCPKERAEISMLEGAVLDIRYGVSRIAYSKDFETLKVDFLSKL
PEMLKMFEDRLCHKTYLNGDHVTHPDFMLYDALDVVLYMDPMCLDAFPKLVCFKKRIEAIP
QIDKYLKSSKYIAWPLQGWQATFGGGDHPPKSDLEVLFQGPLGSPNSRVDMRTSYTVTLP
EDPPAAPFPALAKELRPRSPLSPSLLLSTFVGLLLNKAKNSKSAQGLAGLRNLGNTCFMNSI
LQCLSNTRELRDYCLQRLYMRDLHHGSNAHTALVEEFAKLIQTIWTSSPNDVVSPSEFKTQI
QRYAPRFVGYNQQDAQEFLRFLLDGLHNEVNRVTLRPKSNPENLDHLPDDEKGRQMWRK
YLEREDSRIGDLFVGQLKSSLTCTDCGYCSTVFDPFWDLSLPIAKRGYPEVTLMDCMRLFT
KEDVLDGDEKPTCCRCRGRKRCIKKFSIQRFPKILVLHLKRFSESRIRTSKLTTFVNFPLRDL
DLREFASENTNHAVYNLYAVSNHSGTTMGGHYTAYCRSPGTGEWHTFNDSSVTPMSSSQ
VRTSDAYLLFYELASPPSRM

His-UCHL1

MGSSHHHHHHSSGLEVLFQGPGSMQLKPMEINPEMLNKVLSRLGVAGQWRFVDVLGLEE
ESLGSVPAPACALLLLFPLTAQHENFRKKQIEELKGQEVSPKVYFMKQTIGNSCGTIGLIHAV
ANNQDKLGFEDGSVLKQFLSETEKMSPEDRAKCFEKNEAIQAAHDAVAQEGQCRVDDKVN
FHFILFNNVDGHLYELDGRMPFPVNHGASSEDTLLKDAAKVCREFTEREQGEVRFSAVALC
KAA

GST-UCHL3

MSPILGYWKIKGLVQPTRLLLEYLEEKYEEHLYERDEGDKWRNKKFELGLEFPNLPYYIDGD
VKLTQSMAIIRYIADKHNMLGGCPKERAEISMLEGAVLDIRYGVSRIAYSKDFETLKVDFLSKL
PEMLKMFEDRLCHKTYLNGDHVTHPDFMLYDALDVVLYMDPMCLDAFPKLVCFKKRIEAIP
QIDKYLKSSKYIAWPLQGWQATFGGGDHPPKSDLEVLFQGPLGSPGIPGSTRAAAMEGQR
WLPLEANPEVTNQFLKQLGLHPNWQFVDVYGMDPELLSMVPRPVCAVLLLFPITEKYEVFR
TEEEEKIKSQGQDVTSSVYFMKQTISNACGTIGLIHAIANNKDKMHFESGSTLKKFLEESVSM
SPEERARYLENYDAIRVTHETSAHEGQTEAPSIDEKVDLHFIALVHVDGHLYELDGRKPFPIN
HGETSDETLLEDAIEVCKKFMERDPDELRFNAIALSAA

GST-UCHL5

MSPILGYWKIKGLVQPTRLLLEYLEEKYEEHLYERDEGDKWRNKKFELGLEFPNLPYYIDGD
VKLTQSMAIIRYIADKHNMLGGCPKERAEISMLEGAVLDIRYGVSRIAYSKDFETLKVDFLSKL
PEMLKMFEDRLCHKTYLNGDHVTHPDFMLYDALDVVLYMDPMCLDAFPKLVCFKKRIEAIP
QIDKYLKSSKYIAWPLQGWQATFGGGDHPPKSDLEVLFQGPLGSMTGNAGEWCLMESDP
GVFTELIKGFGCRGAQVEEIWSLEPENFEKLKPVHGLIFLFKWQPGEEPAGSVVQDSRLDTI
FFAKQVINNACATQAIVSVLLNCTHQDVHLGETLSEFKEFSQSFDAAMKGLALSNSDVIRQV
HNSFARQQMFEFDTKTSAKEEDAFHFVSYVPVNGRLYELDGLREGPIDLGACNQDDWISA
VRPVIEKRIQKYSEGEIRFNLMAIVSDRKMIYEQKIAELQRQLAEEPMDTDQGNSMLSAIQSE
VAKNQMLIEEEVQKLKRYKIENIRRKHNYLPFIMELLKTLAEHQQLIPLVEKAKEKQNAKKAQ
ETK

GST-BAP1

MSPILGYWKIKGLVQPTRLLLEYLEEKYEEHLYERDEGDKWRNKKFELGLEFPNLPYYIDGD
VKLTQSMAIIRYIADKHNMLGGCPKERAEISMLEGAVLDIRYGVSRIAYSKDFETLKVDFLSKL
PEMLKMFEDRLCHKTYLNGDHVTHPDFMLYDALDVVLYMDPMCLDAFPKLVCFKKRIEAIP
QIDKYLKSSKYIAWPLQGWQATFGGGDHPPKSDLEVLFQGPLGSMNKGWLELESDPGLFT
LLVEDFGVKGVQVEEIYDLQSKCQGPVYGFIFLFKWIEERRSRRKVSTLVDDTSVIDDDIVNN
MFFAHQLIPNSCATHALLSVLLNCSSVDLGPTLSRMKDFTKGFSPESKGYAIGNAPELAKAH
NSHARPEPRHLPEKQNGLSAVRTMEAFHFVSYVPITGRLFELDGLKVYPIDHGPWGEDEE
WTDKARRVIMERIGLATAGEPYHDIRFNLMAVVPDRRIKYEARLHVLKVNRQTVLEALQQLI
RVTQPELIQTHKSQESQLPEESKSASNKSPLVLEANRAPAASEGNHTDGAEEAAGSCAQA
PSHSPPNKPKLVVKPPGSSLNGVHPNPTPIVQRLPAFLDNHNYAKSPMQEEEDLAAGVGR
SRVPVRPPQQYSDDEDDYEDDEEDDVQNTNSALRYKGKGTGKPGALSGSADGQLSVLQP
NTINVLAEKLKESQKDLSIPLSIKTSSGAGSPAVAVPTHSQPSPTPSNESTDTASEIGSAFNS
PLRSPIRSANPTRPSSPVTSHISKVLFGEDDSLLRVDCIRYNRAVRDLGPVISTGLLHLAEDG
VLSPLALTEGGKGSSPSIRPIQGSQGSSSPVEKEVVEATDSREKTGMVRPGEPLSGEKYSP
KELLALLKCVEAEIANYEACLKEEVEKRKKFKIDDQRRTHNYDEFICTFISMLAQEGMLANLV
EQNISVRRRQGVSIGRLHKQRKPDRRKRSRPYKAKRQ
